# Risk Factors for Poor Treatment Outcomes in Patients with MDR-TB and XDR-TB in China: Retrospective Multi-Center Investigation

**DOI:** 10.1371/journal.pone.0082943

**Published:** 2013-12-05

**Authors:** Shenjie Tang, Shouyong Tan, Lan Yao, Fujian Li, Li Li, Xinzhi Guo, Yidian Liu, Xiaohui Hao, Yanqiong Li, Xiuxiu Ding, Zhanjun Zhang, Li Tong, Jianan Huang

**Affiliations:** 1 Department of Respiratory Medicine, The First Affiliated Hospital of Soochow University, Suzhou, China; 2 Tuberculosis Center for Diagnosis and Treatment, Shanghai Pulmonary Hospital, Tongji University School of Medicine, Shanghai, China; 3 Department of Tuberculosis, Guangzhou Chest Hospital, State Key Laboratory of Respiratory Disease, Guangzhou, China; 4 Hangzhou Red Cross Hospital, Hangzhou, China; 5 Tianjin Haihe Hospital, Tianjin, China; 6 Henan Infectious Hospital, Zhengzhou, China; University of Cape Town, South Africa

## Abstract

**Background:**

The treatment of patients with MDR- and XDR-TB is usually more complex, toxic and costly and less effective than treatment of other forms of TB. However, there is little information available on risk factors for poor outcomes in patients with MDR- and XDR-TB in China.

**Methodology/Principal Findings:**

We retrospectively analyzed the clinical records of HIV-negative TB Patients with culture-proven MDR- or XDR-TB who were registered from July 2006 to June 2011 at five large-scale Tuberculosis Specialized Hospitals in China. Among 1662 HIV-seronegative TB cases which were culture-positive for M. tuberculosis complex and had positive sputum-smear microscopy results, 965 cases (58.1%) were DR-TB, and 586 cases (35.3%) were classified as having MDR-TB, accounting for 60.7% of DR-TB. 169 cases (10.2%) were XDR-TB, accounting for 17.5% of DR-TB, 28.8% of MDR-TB. The MDR-TB patients were divided into XDR-TB group (n=169) and other MDR-TB group (non-XDR MDR-TB) (n=417). In total, 240 patients (40.95%) had treatment success, and 346 (59.05%) had poor treatment outcomes. The treatment success rate in other MDR-TB group was 52.2%, significantly higher than that in the XDR-TB group (13%, *P*<0.001). In multivariate logistic regression analysis, poor outcomes were associated with duration of previous anti-TB treatment of more than one year (OR, 0.077; 95% CI, 0.011-0.499, P<0.001), a BMI less than 18.5 kg/m^2^ (OR, 2.185; 95% CI, 1.372-3.478, P<0.001), XDR (OR, 13.368; 95% CI, 6.745-26.497, P<0.001), retreatment (OR, 0.171; 95% CI, 0.093-0.314, P<0.001), diabetes (OR, 0.305; 95% CI, 0.140-0.663, P=0.003), tumor (OR, 0.095; 95% CI, 0.011-0.795, P=0.03), decreased albumin (OR, 0.181; 95% CI, 0.118-0.295, P<0.001), cavitation (OR, 0.175; 95% CI, 0.108-0.286, P<0.001).

**Conclusions/Significance:**

The patients with MDR-TB and XDR-TB have poor treatment outcomes in China.The presence of extensive drug resistance, low BMI, hypoalbuminemia, comorbidity, cavitary disease and previous anti-TB treatment are independent prognostic factors for poor outcome in patients with MDR-TB.

## Introduction

Despite global efforts to control tuberculosis (TB), the global burden of TB remains enormous. There were estimated to be 8.7 million incident cases of TB in 2011. There were also 1.4 million deaths from TB [1]. The increasing incidence of multidrug resistant tuberculosis (MDR-TB) and extensively drug-resistant (XDR-TB) tuberculosis is a major concern for TB control programs worldwide [2]. MDR-TB is defined as TB with resistance to both isoniazid and rifampin. XDR-TB is defined as TB with resistance to at least isoniazid, rifampin, a fluoroquinolone, and 1 of 3 injectable second-line drugs (amikacin, kanamycin, or capreomycin). According to WHO, 3.7% (2.1–5.2%) of new cases and 20% (13–26%) of previously treated cases are estimated to have MDR-TB. By the end of 2011, XDR-TB had been identified in 84 countries globally [1].

China is one of the world’s 22 countries with the highest burden of tuberculosis. China is also one of the world’s 27 countries with the highest burden of MDR-TB/XDR-TB. The prevalence of drug resistant tuberculosis is very high [3]. According to a national survey of drug-resistant tuberculosis in China, 5.7% of new cases and 25.6% of previously treated cases had MDR-TB. Approximately 8% of the patients with MDR-TB had XDR-TB [4]. According to the results from nationwide population-based survey of TB in 2010 that only included those aged ≥15 years, the prevalence of TB was 459 cases per 100,000 population. The total rate of drug resistance was 42.1%. The frequency of MDR-TB among pulmonary TB patients in China was 6.8%. The proportion of MDR-TB cases with XDR-TB was 23.59% [5]. The study from Shandong showed that 3.6% (95%CI, 2.9%-4.6%) was multidrug-resistant (MDR) isolates, and 20.0% (95%CI, 11.9%-31.4%) of patients with MDR-TB were XDR [6]. The prevalence of MDR- and XDR-TB is of serious concern in China.

The treatment of patients with XDR-TB is usually more complex, toxic and costly and less effective than treatment of other forms of TB because of the severity of XDR-TB disease and shortage of efficacious anti-TB drugs [7]. However, there is little information available on treatment outcome of patients with XDR-TB in China. To investigate this issue, we performed a large retrospective multi-center survey to determine the characteristics, treatment outcomes and risk factors associated with poor treatment outcome among patients with MDR- and XDR-TB in China.

## Methods

### Ethical Approval

Individual participants gave written informed consent before enrollment in the study. All the patient information was routinely collected and recorded by attending physicians. The study was approved by The Ethics Committee of five large-scale Tuberculosis Specialized Hospitals, China.

### Setting and Study population

All patients with culture-proven MDR-TB registered from July 2006 to June 2011 in five large-scale Specialized Tuberculosis Hospitals in China were retrospectively evaluated. The five hospitals are situated in the south, north, east and center of China. All of them are provincial diagnosis and treatment centers for TB, having 3000 beds in total. Because all TB patients receive HIV testing at diagnosis and HIV-infected patients are treated at separate facilities, all subjects in this report were HIV-seronegative. All patients were initially treated as inpatients, but some persons were followed on an outpatient basis after hospitalization. Medical records from in-patient and out-patient treatments were reviewed for patients’ demographics, TB treatment history, comorbidities, drug susceptibility test (DST) results, chest radiographs, treatment modalities and outcomes. According to previous studies [7-11] and our clinical experience, the comorbidities including diabetes, COPD, Chronic hepatitis, tumor, hepatic dysfunction and decreased albumin could be risk factors associated with poor treatment outcome among patients with MDR- and XDR-TB. Thus they were finally selected as variables.

### M. tuberculosis cultures and drug susceptibility testing

Sputum for this study was routinely tested by optical microscopic analysis of Ziehl-Nielsen–stained smears and by culture on Lowenstein-Jensen medium and the BACTEC Mycobacterial Growth Indicator Tube (MGIT) 960 system (Becton Dickinson Diagnostic Systems, Sparks, MD, USA). Drug susceptibility testing (DST) of positive cultures was performed using the MGIT 960 System at the following concentrations: 0.1μg/mL isoniazid, 1 μg/mL rifampin, 5 μg/mL ethambutol, 1μg/mL streptomycin, 2.5 μg/mL capreomycin, 1μg/mL amikacin, 2 μg/mL ofloxacin [12]. All the tests were performed at the TB reference laboratory, and quality control was routinely performed.

### Definitions

New TB cases were defined as TB patients whose medical records indicated that the patient had denied having any prior anti-TB treatment or any history of more than 30 days of anti-TB treatment. Retreatment TB cases were TB patients who had been receiving TB treatment for at least 30 days or who had documented evidence of prior treatment in the case report or surveillance database [13,14]. MDR-TB was defined as TB with resistance to at least isoniazid and rifampin. XDR-TB was defined as TB with resistance to at least isoniazid, rifampin, a fluoroquinolone (e.g. ofloxacin, levofloxacin) and one of three injectable second-line drugs (capreomycin, kanamycin, and amikacin)[13,14]. Treatment outcomes were defined according to the WHO and International Union Against Tuberculosis and Lung Disease (IUATLD) guidelines [13-15]. A "Cured" patient was defined as one who had completed treatment according to the program protocol and had been consistently culture-negative (with at least five results) for the final 12 months of treatment for TB. A patient who "Completed Treatment" was defined as a one who had completed treatment according to program protocol but did not meet the definition for cured because of lack of bacteriological results. The category "Died" comprised any patient who died for any reason during the course of TB treatment. "Treatment Failure" was recorded if two or more of the five cultures recorded in the final 12 months of therapy were positive, or if any one of the final three cultures was positive. The "Defaulted" category comprised any patient whose TB treatment was interrupted for 2 or more consecutive months for any reason. The "Transferred Out" category comprised any patient who had been transferred to another reporting and recording unit and for whom the treatment outcome was unknown. For analysis purposes, Cured and Completed Treatment outcomes were combined as "Treatment Success", whereas others were combined as "Poor Treatment Outcome".

### Regimen and management

Physicians designed individualized treatment regimens for patients with MDR-TB on the basis of DST results and history of drugs taken by the patients [13,14]. Each regimen contained at least four drugs to which the culture was susceptible or probably susceptible. More than five or six drugs might have been started if extensive, bilateral pulmonary disease presented. Anti-tuberculosis drugs mainly included an injectable agent (amikacin or capreomycin), a quinolone (levofloxacin, gatifloxacin, moxifloxacin), para-aminosalicylic acid, prothionamide, pyrazinamide, clarithromycin, ethambutol, etc. An injectable agent was used for a minimum of six months, or 12 months for XDR-TB, for the initial intensive phase, and the total duration was 24 months [13,14]. Each patient was given directly observed therapy (DOT) throughout treatment. DOT was carried out in hospital by trained medical staff, or in the case of out-patients, by trained family members or supervisors in the community.

### Statistical analysis

All statistical analyses were performed using SPSS14.0 software, version 14.0 (SPSS, Chicago). Comparisons of categorical variables were performed using the Pearson Chi-square tests or Fisher’s exact tests to compare different groups. Comparisons of continuous variables were performed using the T tests to compare different groups.To evaluate the predictors for poor treatment outcome, we compared selected clinical variables between the treatment success and poor treatment outcome groups, using univariate comparison and subsequent multiple logistic regression. In regression, stepwise and backward selection procedures were used to select statistically significant variables to be maintained in the final model. The probability criterion by which a variable was entered and retained was 0.05. Interaction terms were assessed by using Wald Chi-Square tests, with p<0.2 considered significant. Statistical significance was set at *P*<0.05.

## Results

### Patient Demographics

Among 1662 HIV-seronegative TB cases which were culture-positive for *M. tuberculosis* complex and had positive sputum-smear microscopy results, 965 cases (58.1%) were drug resistant (DR), and 586 cases (35.3%) were classified as having MDR-TB, accounting for 60.7% of DR-TB. 169 cases (10.2%) were XDR-TB, accounting for 17.5% of DR-TB, 28.8% of MDR-TB. The patients were divided into XDR-TB group (n=169) and Other MDR-TB group (non-XDR MDR-TB) (n=417).The percentage of females in the XDR group was 39.1%, significantly higher than in the Other MDR group (30.0%, *P*=0.034). Median ages in XDR and Other MDR groups were 51.0 ± 15.68 and 44.4 ± 15.05 years, respectively, and the difference was significant ( *P*<0.001) (table 1).

**Table 1:** Demographics of patients with XDR-TB and Other MDR-TB.

**Demographics**	**XDR**	**Other MDR**	***P* value**
	(n=169)	(n=417)	
	No. (%)	No. (%)	
Male	103(60.9)	292(70.0)	0.034
Female	66(39.1)	125(30.0)	
Smoking	37(21.9)	128(30.7)	0.032
Rural	86(50.9)	221(53.0)	0.643
Urban	83(49.1)	196(47.0)	
Education*			0.112
Illiteracy	8( 4.7)	7( 1.7)	
Primary school	42(24.9)	88(21.1)	
Middle school	94(55.6)	261(62.6)	
Undergraduate	25(14.8)	61(14.6)	
Occupation			0.953
Peasant	54(32.0)	132(31.7)	
Worker	68(40.2)	175(42.0)	
Civil servant	7( 4.1)	13( 3.1)	
Attendant	24(14.2)	54(12.9)	
Other occupation	16( 9.5)	43(10.3)	
Mean age, y ±SD (range)	51.0± 15.68(21~84)	44.4± 15.05 (14~88)	<0.001
Mean BMI ±SD (range)	19.1± 2.79(12~30)	19.2± 2.62(12~30)	0.597

NOTE. BMI, body mass index (calculated as the weight in kilograms divided by the square of the height in meters); * Determined by Fisher’s exact test, Not indicated: Determined by Pearson’s Chi-square test.

### Clinical characteristics

Among the 586 MDR-TB cases, retreated patients accounted for 78.67% (461/586), and there was no significant difference in retreatment between XDR and Other MDR groups (*P*=0.815). There was a much higher proportion of patients with comorbidity, decreased albumin, and cavitary disease among the patients in the XDR group compared to patients in the Other MDR group (*P*=0.001). The course of disease in the XDR group was 7.2±8.75 years, significantly higher than that in the Other MDR group (*P*=0.004). More detailed information on clinical characteristics is shown in Table 2.

**Table 2:** Clinical characteristics of patients with XDR-TB and Other MDR-TB.

**Clinical characteristics**	**XDR**	**Other MDR**	***P* value**
	(n=169)	(n=417)	
	No. (%)	No. (%)	
New case	35(20.7)	90(21.6)	0.815
Retreatment case	134(79.3)	327(78.4)	
With comorbidity	64(37.9)	102(24.5)	0.001
Diabetes	30(17.8)	50(12.0)	0.066
COPD	21(12.4)	29( 7.0)	0.032
Chronic hepatitis	13( 7.7)	23( 5.5)	0.320
Tumor*	5( 3.0)	7( 1.7)	0.341
Hepatic dysfunction	21(12.4)	49(11.8)	0.819
Decreased albumin	61(36.1)	79(18.9)	0.001
With cavitation	130(76.9)	259(62.1)	0.001
Course of disease, y±SD (range)	7.2±8.75(1~50)	5.1±5.55(1~40)	0.004
Duration of earlier treatment, y±SD (range)	2.8±2.83(1~20)	2.4±1.61(0.5~10)	0.063

NOTE. COPD, chronic obstructive pulmonary disease; * Determined by Fisher’s exact test, and the p value for the “range of cavitation” variable is calculated on the subset of patients with cavitation; Not indicated: Determined by Pearson’s Chi-square test.

### Drug Resistance Rate

For any drug, except isoniazid and rifampicin, the proportion of patients with cultures that were drug-resistant was significantly higher in patients with XDR-TB than in the Other MDR-TB patients (*P*<0.001) (Table 3). The proportions resistant to streptomycin, ethambutol, ofloxacin, capreomycin and amikacin in all 586 MDR patients were 80.5%, 80.5%, 69.3%, 31.1% and 29.7%, respectively.

**Table 3:** Frequency of drug resistance among patients with XDR-TB and Other MDR-TB.

**TB Drugs**	**XDR**	**Other MDR**	**Total**	***P* value**
	(n=169)	(n=417)	(n=586)	
	No. (%)	No. (%)	No. (%)	
Isoniazid	169(100)	417(100)	586(100)	
Rifampicin	169(100)	417(100)	586(100)	
Streptomycin	156(92.3)	316(75.8)	472(80.5)	<0.001
Ethambutol	150(88.8)	322(77.2)	472(80.5)	0.001
Ofloxacin	169(100)	237(56.8)	406(69.3)	<0.001
Capreomycin	140(82.8)	42(10.1)	182(31.1)	<0.001
Amikacin	135(79.9)	39(9.4)	174(29.7)	<0.001

### Treatment Outcomes and Predictors of Poor Treatment Outcomes

The assessment of treatment outcomes revealed that 92 patients (15.7%) were Cured, 148 (25.25%) Completed Therapy, 22 (3.75%) Died, 280 (47.78%) experienced Treatment Failure, 32 (5.46%) Defaulted treatment, and 12 (2.05%) were Transferred Out. In total, 240 patients (40.95%) had Treatment Success, and 346 (59.05%) had Poor Treatment Outcomes. The Treatment Success rate in the Other MDR-TB group was 52.2%, significantly higher than that in the XDR-TB group (13%, *P*<0.001), whereas Poor Treatment Outcomes were more common in patients with XDR-TB than in patients with Other MDR-TB (87 vs.47.8%; *P*<0.001) (Table 4).

**Table 4:** Treatment Outcomes of patients with XDR-TB and Other MDR-TB.

**Treatment Outcomes**	**XDR**	**Other MDR**	**Total**	***P* value**
	(n=169)	(n=417)	(n=586)	
	No. (%)	No. (%)	No. (%)	
Treatment Success	22(13)	218(52.2)	240(40.95)	<0.001
Cure	8(4.7)	84(20.1)	92(15.7)	<0.001
Treatment Completion	14(8.3)	134(32.1)	148(25.25)	<0.001
Poor Treatment Outcome	147(87)	199(47.8)	346(59.05)	<0.001
Death	8(4.7)	14(3.4)	22(3.75)	0.427
Failure	124(73.4)	156(37.4)	280(47.78)	<0.001
Default	10(5.9)	22(5.3)	32(5.46)	0.757
Transfer Out	5(3.0)	7(1.7)	12(2.05)	0.322

In multivariate logistic regression analysis, Poor Treatment Outcomes were associated with duration of previous anti-TB treatment of more than one year (OR, 0.077; 95% CI, 0.011-0.499, P<0.001), a BMI less than 18.5 kg/m^2^ (OR, 2.185; 95% CI, 1.372-3.478, P<0.001), XDR (OR, 13.368; 95% CI, 6.745-26.497, P<0.001), retreatment (OR, 0.171; 95% CI, 0.093-0.314, P<0.001), diabetes (OR, 0.305; 95% CI, 0.140-0.663, P=0.003), tumor (OR, 0.095; 95% CI, 0.011-0.795, P=0.03), decreased albumin (OR, 0.181; 95% CI, 0.118-0.295, P<0.001), cavitation (OR, 0.175; 95% CI, 0.108-0.286, P<0.001) (Table 5). In multivariate logistic regression analysis, having a tumor was the only independent risk factor associated with death (OR, 23.584; 95% CI, 5.361-103.737, P<0.0001).

**Table 5:** Predictors of Poor Treatment Outcomes for 586 patients with MDR-TB.

**Variables**	**Treatment Success (n=240)**	**Poor Treatment Outcome (n=346)**	**Univariate analysis: p-value**	**Multivariate logistic regression**	
	**No. (%)**	**No. (%)**		**OR (95% CI)**	**p-value**
Male Sex	170(70.8)	225(65.0)	0.140		
Age group			0.027		
Age<45	129(53.8)	151(43.6)			
45≤Age≤65	82(34.2)	131(37.9)			
Age>65	29(12.1)	64(18.5)			
Course of disease			<0.001		
<1 year	89(37.08)	83(23.99)			
≥1 year	151(62.92)	263(76.01)			
Duration of previous anti-TB treatment			<0.001		
<1 year	93(38.75)	66(19.1)			
≥1year	147(61.25)	244(80.9)		0.077(0.011-0.499)	<0.001
BMI			<0.001		
BMI <18.5	78(32.5)	172(49.7)		2.185(1.372-3.478)	<0.001
BMI≥18.5	162(67.5)	174(50.3)			
Smoking	70(29.2)	95(27.5)	0.651		
Residence					
Rural	118(49.2)	189(54.6)	0.193		
Urban	122(50.8)	157(45.4)			
Education			0.663		
Illiteracy	5(2.1)	10(2.9)			
Primary school	53(22.1)	77(22.3)			
Middle school	151(62.9)	204(58.9)			
Undergraduate	31(12.9)	55(15.9)			
Occupation			0.391		
Peasant	66(27.5)	120(34.7)			
Worker	108(45.0)	135(39.0)			
Civil servant	7(2.9)	13(3.8)			
Attendant	33(13.8)	45(13.0)			
Other occupation	26(10.8)	33( 9.5)			
Group			<0.001		
Other MDR	218(90.8)	199(57.5)			
XDR	22(9.2)	147(42.5)		13.368(6.745-26.497)	<0.001
Chemotherapy history			<0.001		
New case	92(38.3)	33(9.5)			
Retreatment case	148(61.7)	313(90.5)		0.171(0.093-0.314)	<0.001
With comorbidity					
Diabetes	15(8.9)	65(15.6)	<0.001	0.305(0.140-0.663)	0.003
COPD	13(7.7)	37(8.9)	0.025		
Chronic hepatitis	6(3.6)	30(7.2)	0.002		
Tumor*	2(1.2)	10(2.4)	0.136	0.095(0.011-0.795)	0.03
Hepatic dysfunction	31(18.3)	39(9.4)	0.546		
Decreased albumin	35(14.6)	105(30.3)	<0.001	0.181(0.118-0.295)	0.001
With cavitation	95(39.6)	294(84.97)	<0.001	0.175(0.108-0.286)	<0.001
Regimen including a later generation quinolone	55(22.9)	63(18.2)	0.162		

NOTE. *Determined by Fisher’s exact test, Not indicated: Determined by Pearson’s Chi-square test.

## Discussion

In our survey, the data from five large-scale Tuberculosis Specialized Hospitals in China showed that 35.3% of cases (586/1662) were classified as having MDR-TB, and 28.8% of MDR-TB was XDR-TB. The percentage of XDR-TB was higher than in the previous surveillance data in China [16-19], in Taiwan [20], and in other countries [9,21,22], and it was similar to the 23.9% which was reported from South Africa in TB and HIV co-infected patients [23-25], similar to the 23.59% from nationwide population-based survey in China [5] and to 20.0% found in the study from Shandong [6]. The high prevalence of XDR-TB could be explained by the fact that our study subjects were from provincial Tuberculosis Specialized Hospitals, where the patients might tend to be selected as being relatively difficult cases. For example, the XDR-TB cases mainly comprised of TB cases with long-term disease (7.2±8.75 years of TB disease, 2.8±2.83 years of previous anti-tuberculosis treatment), cavitary diseases (76.9%); co-morbid diabetes mellitus (17.8%) Moreover, 79.3% were retreatment cases. This picture can be taken as indicative of the current situation of patients with tuberculosis in Tuberculosis Specialized Hospitals in China. Although this survey was not conducted in communities, it may also provide an objective reflection of the high prevalence of XDR-TB in China in general because in China most patients with TB are treated in Tuberculosis Specialized Hospitals. In our study, the percentage of females in the XDR group was 39.1%, significantly higher than in the Other MDR group (*P*=0.034), and similar to a study in Korea and in South Africa [21,26]. Why there are higher percentages of females among XDR patients than Other MDR patients remains unknown and should be further investigated.

In our study, the proportion of all MDR-TB patients with cultures resistant to ofloxacin was up to 69.3% in all MDR-TB patients, and the drug resistant proportion of ofloxacin was 56.8% in Other MDR groups, higher than 6.4%, 16.6% and 42.8% which were respectively reported from Latvia [27], South Korea [10] and Tai Wan [28]. Two reasons might contribute to the high drug resistant proportions: First, fluoroquinolones have been widely used for the treatment of respiratory tract bacterial infection because of their better effects and slight adverse reaction. Second, we also prescribe fluoroquinolones for drug resistant TB patients and some drug susceptible TB patients who can’t tolerate first line anti-tuberculosis drugs. Our study also indicated that more than 80% of XDR-TB patients were resistant to drugs such as streptomycin, ethambutol, capreomycin and amikacin, in addition to resistance to isoniazid and rifamplicin, as well as ofloxacin. This is significantly higher than the percentages from other studies [21,28]. In China, the above seven drugs are the most commonly used ones to treat TB and DR-TB. Giventhe existing scarcity of effective drugs, such a wide range of resistance complicates selection of drugs for the treatment of XDR-TB.

Differences regarding the treatment success rates for MDR and XDR-TB patients are reported in the literature. In a study conducted in Iran, the success rates of MDR in total, non-XDR MDR and XDR were 76.5% (39/51), 87.2% (34/39) and 41.7% (5/12), respectively [29]. According to the results of a recent meta-analysis of 13 observational studies, treatment of XDR tuberculosis succeeded in 43.7% of cases [7]. In our study, the overall treatment success rate (40.95%) of patients with all MDR-TB (including XDR-TB) in our study was a little lower than that in two South Korean reports (45.3%-48.2%) [10,30] and in another report in China (51.4%) [31], but much lower than the rates of 60.4%-72.6% in other reports [11,25,32]. Meanwhile, the 13% treatment success rate of patients with XDR-TB in our study was much lower than the 29.2-60.4% rates in other studies [10,11,27,31,32]. In addition, treatment success rate of the patients with XDR-TB in our study was much lower than that of the patients with Other MDR-TB (excluding XDR-TB) in our study. We also found that in contrast to the poor treatment outcome, the mortality rate of XDR-TB was 4.7 %, much lower than those reported by a few other studies [7,11,33]. Most patients can live long, but had protracted infectious period, similar to other publications of HIV-seronegative cohorts with XDR-TB [11,31,34]. In China, the treatment success rate of MDR- and XDR-TB is low. This can be ascribed to the following important factors: firstly, shortages of second-line anti-tuberculosis drugs are experienced in many settings, including major cities in China. Consequently, the selection of available effective drugs for MDR- and XDR-TB treatment is even more limited and often restricting treatment regimens to less than the WHO-recommended four effective drugs [13]. Secondly, because treatment costs for MDR- and XDR-TB was very high and free second-line TB drugs are not routinely available in China, many patients with MDR- and XDR-TB in China could not afford the cost of treatment, and lead to intermittent or irregular treatment. Thirdly, to manage the patients with MDR- and XDR-TB is very difficult in China. Because of poverty, the patients with MDR- and XDR-TB in rural areas have to leave their hometowns and work in other cities far away. Some of them are people of no fixed habitation. Therefore, these patients cannot receive effective treatment management, which leads to intermittent or irregular treatment. Lastly, mycobacteria culture and drug susceptibility testing (DST) cannot be performed in most laboratories in China. Many MDR- and XDR-TB cases are unlikely to be found as early as they should be. Thus, patients cannot be treated as early as possible, which has a great impact on treatment effectiveness.

It is well known that previous anti-TB treatment is the strongest risk factor for being ill with MDR-TB [35,36]. In multivariate logistic regression analysis, we found that poor treatment outcomes were associated with duration of previous anti-TB treatment of more than one year (OR, 0.077; 95% CI, 0.011-0.499, P<0.001). This is in line with the results from previous studies [10,32]. Dheda et al. [33] found that previous treatment for MDR-TB was an independent predictor of death. Possible reasons for the poor treatment outcomes for the poor treatment outcomes include the long duration of drug-susceptible and MDR-TB treatment before initiation of XDR treatment, as well as further delays between XDR diagnosis and treatment initiation.

According to the results of a recent meta-analysis, low BMI is a risk factor for worse outcome in MDR- and XDR-TB patients [7,8]. In multivariate logistic regression analysis, poor treatment outcomes were associated with a BMI less than 18.5 kg/m^2^ (OR, 2.185; 95% CI, 1.372-3.478, P<0.001). Furthermore, decreased levels of serum albumin were associated with poor treatment outcomes. A previous study showed that a higher level of serum albumin was inversely related to treatment failure in patients with XDR-TB [11]. Hypoalbuminemia and malnutrition could impair host immunity against Mycobacterium tuberculosis. China is a developing country, and its economic development is uneven. In our study, most patients with MDR- and XDR-TB are peasants and workers, some of whom have under-nutrition due to lack of money. In another study analyzing predictors of poor treatment outcome in multi- and extensively drug-resistant pulmonary TB in Estonia, underlying comorbidities (adjusted OR, 2.62; 95% CI, 1.00-6.87) were independent risk factors for treatment failure [11]. We found that diabetes was also an independent predictor of poor treatment outcomes. Some patients with diabetes have under-nutrition. Thus nutritional status is an important predictor of treatment outcomes of patients with MDR- and XDR-TB.

Several studies have suggested an association between XDR-TB and poor treatment outcomes in MDR-TB patients [10,27,29,37]. We also observed that XDR was an independent predictor of poor treatment outcomes (OR, 13.368; 95% CI, 6.745-26.497, P<0.001). In another study conducted in South Korea, multivariate analysis showed that XDR-TB was the strongest negative predictor of treatment success and probably contributes to the low rate of treatment success in patients with MDR-TB [10]. We found that cavitary disease (OR, 0.175; 95% CI, 0.108-0.286, P<0.001) was an independent predictor of poor treatment outcomes, similar to previous studies [10,27].

Previous studies have also suggested an association between resistance to fluoroquinolones and poor treatment outcomes in MDR-TB patients [29,32,37], this was observed in our study (data not shown). Some studies showed that later-generation fluoroquinolones such as moxifloxacin may improve treatment outcomes for patients with MDR- and XDR-TB [7,37]. However, some discordant results were reported in the literature. Lee and his colleagues [38] reported that the treatment success rate was 78.9% in the levofloxacin group and 83.3% in the moxifloxacin group, and there was no significance between the two group (p = 0.42). Another study underscores current WHO recommendations to use moxifloxacin when there is resistance to early-generation fluoroquinolones such as ofloxacin, restricting this recommendation to strains with moxifloxacin MICs of less than or equal to 2µg/ml [39]. We did not find any significant association between treatment success and regimen including a later generation quinolone. A high resistance proportion (13/30, 43.33%) of moxifloxacin in MDR-TB patients was observed in another study of ours despite a lower resistance proportion (5/51, 9.8%) of moxifloxacin in sensitive-TB patients (unpublished data).These results suggest that moxifloxacin does not improve treatment outcomes in the cohort collectively. Cameniero et al. [40] suggested the use of high-dose levofloxacin.

Our study has several limitations. Firstly, the number of hospitals participating in the study was not big enough although this was a multi-center survey. Thus, the findings cannot fully represent the clinical characteristic and current situation of treatment of XDR-TB in China, and the findings may represent TB in specialized TB hospitals, but not in the population as a whole. Secondly, this study was limited by the relatively small number of XDR-TB cases. A larger sample can better reflect the treatment outcomes and its predictors of MDR- and XDR-TB. Thirdly, long-term follow-up of the treatment outcomes was not conducted in this study. Long-term clinical efficacy in these patients needs to be investigated to further understand recurrence rates of MDR- and XDR-TB. Fourthly, HIV-infected tuberculosis patients were not included in our study since HIV-infected tuberculosis patients are not admitted to our Tuberculosis Specialized Hospitals in China. All patients are tested for HIV in China. If he is HIV-positive he will be transferred to HIV Specialized hospital immediately. So this study could not show the profile of MDR-TB and XDR-TB in China.

Despite these limitations, to our knowledge, this might be the first multi-center investigation to assess the characteristics and risk factors associated with poor treatment outcomes among patients with MDR- and XDR-TB in China. Our study demonstrated that the patients with MDR-TB and XDR-TB have poor treatment outcomes in China, and the presence of extensive drug resistance, low BMI, hypoalbuminemia, comorbidity, cavitary disease and previous anti-TB treatment are independent prognostic factors for poor outcome in patients with MDR-TB. Effective TB control strategies should be implemented to prevent the development and spread of MDR- and XDR-TB in China. Economical and rapid diagnostic methods should be developed for timely identification of MDR-TB or XDR-TB, especially in patients with a history of TB and prior anti-TB treatment. Greater measures should be taken to provide adequate and free treatment for patients with MDR- and XDR-TB. In addition, more intensive efforts should be made to manage MDR- and XDR-TB cases more effectively to improve their treatment outcomes.
